# Optimizing labelling conditions of ^213^Bi-DOTATATE for preclinical applications of peptide receptor targeted alpha therapy

**DOI:** 10.1186/s41181-016-0014-4

**Published:** 2016-05-14

**Authors:** Ho Sze Chan, Erik de Blois, Mark W. Konijnenberg, Alfred Morgenstern, Frank Bruchertseifer, Jeffrey P. Norenberg, Fred J. Verzijlbergen, Marion de Jong, Wouter A. P. Breeman

**Affiliations:** 1 Erasmus MC, Department of Radiology and Nuclear Medicine, ‘s-Gravendijkwal 230, 3015 CE Rotterdam, The Netherlands; 2 European Commission, Joint Research Centre, Institute for Transuranium Elements (ITU), Hermann-von-Helmholtz-Platz 1, Eggenstein-Leopoldshafen, 76344 Karlsruhe, Germany; 3 Radiopharmaceutical Sciences Program, College of Pharmacy, University of New Mexico Health Sciences Center, Albuquerque, 87131-0001 NM USA

**Keywords:** Somatostatin, ^213^Bismuth, Targeted alpha therapy, Radiochemical purity, Ascorbic acid, Absorbed dose

## Abstract

**Background:**

^213^Bismuth (^213^Bi, T_1/2_ = 45.6 min) is one of the most frequently used α-emitters in cancer research. High specific activity radioligands are required for peptide receptor radionuclide therapy. The use of generators containing less than 222 MBq ^225^Ac (actinium), due to limited availability and the high cost to produce large-scale ^225^Ac/^213^Bi generators, might complicate in vitro and in vivo applications though.

Here we present optimized labelling conditions of a DOTA-peptide with an ^225^Ac/^213^Bi generator (< 222 MBq) for preclinical applications using DOTA-Tyr^3^-octreotate (DOTATATE), a somatostatin analogue. The following labelling conditions of DOTATATE with ^213^Bi were investigated; peptide mass was varied from 1.7 to 7.0 nmol, concentration of TRIS buffer from 0.15 mol.L^-1^ to 0.34 mol.L^-1^, and ascorbic acid from 0 to 71 mmol.L^-1^ in 800 μL. All reactions were performed at 95 °C for 5 min. After incubation, DTPA (50 nmol) was added to stop the labelling reaction. Besides optimizing the labelling conditions, incorporation yield was determined by ITLC-SG and radiochemical purity (RCP) was monitored by RP-HPLC up to 120 min after labelling. Dosimetry studies in the reaction vial were performed using Monte Carlo and in vitro clonogenic assay was performed with a rat pancreatic tumour cell line, CA20948.

**Results:**

At least 3.5 nmol DOTATATE was required to obtain incorporation ≥ 99 % with 100 MBq ^213^Bi (at optimized pH conditions, pH 8.3 with 0.15 mol.L^-1^ TRIS) in a reaction volume of 800 μL. The cumulative absorbed dose in the reaction vial was 230 Gy/100 MBq in 30 min. A minimal final concentration of 0.9 mmol.L^-1^ ascorbic acid was required for ~100 MBq (t = 0) to minimize radiation damage of DOTATATE. The osmolarity was decreased to 0.45 Osmol/L.

Under optimized labelling conditions, ^213^Bi-DOTATATE remained stable up to 2 h after labelling, RCP was ≥ 85 %. In vitro showed a negative correlation between ascorbic acid concentration and cell survival.

**Conclusion:**

^213^Bismuth-DOTA-peptide labelling conditions including peptide amount, quencher and pH were optimized to meet the requirements needed for preclinical applications in peptide receptor radionuclide therapy.

**Electronic supplementary material:**

The online version of this article (doi:10.1186/s41181-016-0014-4) contains supplementary material, which is available to authorized users.

## Background

Peptide receptor radionuclide therapy (PRRT) is an effective treatment for metastatic and inoperable neuroendocrine tumours (van der Zwan et al. 2015; Bergsma et al. 2012; Bodei et al. 2012). The most common radionuclides used for PRRT include ^177^Lu (β^−^-emitter, T_1/2_ = 6.71 days, 149 and 497 keV) and ^90^Y (β^−^-emitter, T_1/2_ = 64.1 h, 935 and 2284 keV).

α-Emitters have been demonstrated to offer an additional treatment option for patients refractory to standard PRRT with β^−^-emitters or chemotherapy (Kratochwil et al. 2011; Jurcic et al. 1995; Cordier et al. 2010). Moreover, α-particles have short path length in tissue (~80 μm), sparing non-target tissues from radiation. ^213^Bi (T_1/2_ = 45.6 min) is an α-emitter and has been applied in several (pre) clinical research studies of targeted alpha therapy (TAT) (Kratochwil et al. 2011; Jurcic et al. 1995; Norenberg et al. 2006; Song et al. 2008; Wild et al. 2011).

PRRT is based on receptor-mediated processes. In order to achieve treatment success, a sufficient cytotoxic dose of radio-peptide must be delivered to the targeted cells (Konijnenberg 2014). Moreover, the number of receptors available on the cell membrane is limited, so high specific activity (SA, expressed in MBq per nmol of peptide) of labelled peptides is advantageous for administration in small animals e.g. mice (~25 g) (Breeman et al. 2001; Breeman et al. 1995; de Jong et al. 1999). Besides high SA, other requirements for preclinical applications include high stability of the radio-peptide at physiological conditions in vitro (~0.3 Osmol.L^−1^, pH ~7.4) and in vivo. In additional, a high osmolarity of the drug-containing solution is inconsistent with maintenance of physiological conditions for in vitro and in vivo applications.

Due to the limited availability and the high cost to produce large-scale ^225^Ac/^213^Bi generators, preclinical studies with ^213^Bi are often being performed using relatively low activity generators (e.g. 222 MBq ^225^Ac). Under these conditions, the standard clinical labelling procedure for ^213^Bi, which was designed for high activity generators (up to 4 GBq) with a reaction volume of 2 mL at > 0.7 Osmol.L^−1^ (Kratochwil et al. 2014), probably needs to be adjusted. Therefore, the labelling conditions for preclinical applications required modifications.

In this study, we systematically studied the consequences using the somatostatin analogue [DOTA^0^, Tyr^3^]-octreotate (DOTATATE) as a model for optimizing labelling conditions of ^213^Bi for preclinical applications, starting from the standard labelling protocol.

Herein we varied the labelling conditions such as peptide amount, quencher and pH. The stability of the labelled peptide and the radiochemical purity (RCP) was monitored up to 2 h after labelling. The absorbed dose rates of ^213^Bi, ^213^Po, ^209^Tl and ^209^Pb (mGy.s^−1^) as function of reaction volume were calculated. The use of the absorbed dose rate is investigated as a possible surrogate indicator for the ionization probability and consequential radiolysis to the peptide in the reaction vial. And lastly, in vitro clonogenic assay was performed to investigate the influence caused by labelling conditions used.

The objective of this study was to establish a practical, ready to use, and reproducible labelling procedure of ^213^Bi-DOTATATE to meet the constraint for the use of peptide as radiopharmaceutical for preclinical applications.

## Methods

### ^213^Bi-elution

The ^225^Ac/^213^Bi generator (222 MBq) was supplied by the Institute for Transuranium Elements (ITU), Germany. Prior to elution, the column was rinsed with diluted HCl (0.01 mol.L^−1^). ^213^Bi was eluted from the ^225^Ac/^213^Bi generator by NaI/HCl (0.1 mol.L^−1^/0.1 mol.L^−1^) in a volume of 600 μL. After elution the column was rinsed and stored with diluted HCl (0.01 mol.L^−1^) (Morgenstern et al. 2012).

### Standard protocol radiolabelling of ^213^Bi-DOTATATE

DOTATATE (DOTA-DPhe-Cys-Tyr-DTrp-Lys-Thr-Cys-Thr, *Mw* 1436 g/mol) was purchased from BioSynthema (St. Louis, MO, USA). All chemicals were purchased from Sigma-Aldrich (Zwijndrecht, the Netherlands).

The following standard labelling protocol was applied: ^213^Bi (600 μL) was added to DOTATATE (7.0 nmol), TRIS buffer (0.34 mol.L^−1^), ascorbic acid (71 mmol.L^−1^) and MilliQ water in a final volume 800 μL at pH 8.7. The reaction was incubated for 5 min at 95 °C. The radionuclide incorporation reaction was halted by cooling the mixture for 2 min in ice and through the addition of 50 nmol diethylenetriaminepentaacetic acid (DTPA, Erasmus MC Pharmacy) to chelate any unbound or “free” ^213^Bi.

### Labelling optimization for preclinical studies

Standard labelling protocol was used as described above, while varying the following conditions:

1. Peptide mass; labelling was performed with 1.7, 3.5 and 7.0 nmol DOTATATE .
2. pH dependence study; 7.0 nmol DOTATATE was labelled in a reaction containing 71 mmol.L^−1^ ascorbic acid and 0.15 mol.L^−1^ and 0.34 mol.L^−1^ TRIS buffer.
3. Quencher dependence study; in absence of ascorbic acid and 0.1, 0.3, 0.9, 2.6, 7.9, 24 and 71 mmol.L^−1^ ascorbic acid.

### Quality control of radiolabelled peptide

The quality control of labelled peptides was determined by ITLC-SG (instant thin layer chromatography silica gel, Varian) and HPLC (high performance liquid chromatography).

Incorporation yield (expressed as mean ± SD) was determined by ITLC-SG using sodium citrate (0.1 mol.L^−1^, pH 5) as mobile phase. The ^213^Bi activity was determined by HPGe detector with a pulse height multichannel analysis (MetorX, Goedereede, The Netherlands and Software Genie 2000 Canberra) at fixed geometry, all measurements were performed for the 440 keV γ-emission by ^213^Bi with a yield of 0.261 per decay. The counting efficiency of the HPGe detector at 440 keV γ-emission was determined using a known amount of ^225^Ac activity provided and calibrated by ITU, Germany (Morgenstern et al. 2012; Ma et al. 2001; McDevitt et al. 1999).

The RCP (expressed as percentage ± SD intact radio-peptide of interest, compared to other detectable radioactive compounds in the same analysis) and stability of the labelled peptide as function of time were determined by HPLC. HPLC-grade methanol and trifluoracetic acid (TFA) were purchased from Mallinckrodt Baker (Deventer, the Netherlands). The HPLC system (Waters 2695 separation module, Alliance, Waters, Etten-Leur, The Netherlands) consisted of a quaternary pump and an autosampler, Waters 2996 photodiode array detector (Waters, Etten-Leur, The Netherlands), radiometric sodium iodide detector (Canberra, Canberra, Genie 2000) and symmetry C_18_ 5 μm column, 4.6 mm ×250 mm (Waters, Etten-Leur, The Netherlands). The mobile phase consisted of buffer A (0.1 % TFA in water) and buffer B (Methanol). The gradient used for the analysis was as described earlier (de Blois et al. 2011). The retention times of the unlabelled DOTATATE and ^213^Bi-DOTATATE were 12.0 ± 0.3 min and 13.0 ± 0.3 min, respectively.

### Dosimetry model of ^213^Bi exposure in reaction vials

The degradation of radiopharmaceuticals by radiolysis is dependent on the amount of energy absorbed within the ligand. Most of the α-particle energy emitted by ^213^Bi and its daughter ^213^Po will be absorbed within the reaction vial with the ^213^Bi-labelled compound, as their particle ranges in water are smaller than the vial dimensions. The energy emitted by the α- and β^−^-particles from ^213^Bi itself and its daughters ^209^Tl and ^209^Pb, however, will not be completely absorbed within the small vials. The activity as a function of time for ^213^Bi, ^213^Po, ^209^ Tl and ^209^Pb was calculated by the Bateman equations, see Additional file 1: Equation 1. The β^−^-emission spectra are summarized in Table 1 together with their ranges in water, from the NIST Star database (http://www.nist.gov/pml/data/star/; assessed 27-11-‘15). The α-particle energies from ^213^Bi and ^213^Po are also indicated with their projected ranges as calculated with the Stopping and Range of Ions in Matter (SRIM version 2013.00 software; www.SRIM.org). The recoil energies from the α-particle emissions are 112 keV (^213^Bi) and 160 keV (^213^Po) (Eckerman KF MIRD2008 2999).

**Table 1:** α- and β-particle emissions by ^213^Bi and its daughters, emission abundances per decay of ^213^Bi or daughter nuclide and energies are from the MIRD Radionuclide data and decay schemes book (Eckerman, 2008). Particle ranges (mm) in water were determined from the NIST Star database (electrons) and with the SRIM code (α-particles). Abundance (Ab.) is expressed in % decay and energy (E) in MeV

α-particles	Ab. (%/decay)	E (MeV)	range (μm)		
^213^Bi	1.94	5.87	46.47		
^213^Po	100	8.38	81.75		
β-particles	Ab. (%/decay)	E_mean_ (MeV)	range (mm)	E_max_ (MeV)	range (mm)
^213^Bi	97.91	0.434	1.45	1.422	6.65
^209^Tl	100	0.655	2.55	1.944	9.48
^209^Pb	100	0.197	0.44	0.644	2.49

**Fig. 1:**
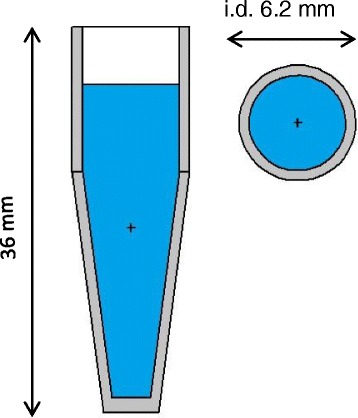
MCNP geometry input for 1 mL reaction vial with 0.8 mL radioactive fluid (in blue), water with density 1 g/mL. The vial wall (in grey) had a thickness of 0.65 mm and was polyethylene with density 0.9 g/mL

Radiation transport was calculated for ^213^Bi containing liquid inside reaction vials to determine the absorbed energy and absorbed dose to the hot liquid. The Monte Carlo codes MCNP5 (MCNP Team The Monte Carlo codes MCNP52005 2999) and MCNPX (Hendricks JS MCNPX Extensions Version 2 5 02005 2999) were used for the calculations. Calculations for the α-particles from ^213^Bi and ^213^Po were performed with MCNPX using the α-particle energies listed in Table 1. The β^−^-spectra and low-energy internal conversion and Auger electron spectra for ^213^Bi, ^209^Tl and ^209^Pb from MIRD Radionuclide data and decay schemes book (Eckerman KF MIRD2008 2999) were used in MCNP5. Particulate radiation emissions with the given energy spectra were simulated so as to be uniformly distributed within a conical reaction vial with isotropic direction emission. All physics processes were taken into account by choosing either the α (MODE A) or the photon–electron mode (MODE P E) and the default PHYS cards with the default cut-off energy at 1 keV for electrons and photons and 4 MeV for α-particles. The *F8 tally determined energy absorption within the hot reaction fluid. Sufficient particle histories (NPS) were used to reduce the variation in the data to be less than 5 % for most cases; NPS was set to 1 × 10^7^ particles. A conically shaped 1 mL reaction vial was modelled with various volumes of radioactive fluid inside the vial. The MCNP-model geometry for a vial containing 0.8 mL liquid is shown in Fig. 1. The labelling volumes were modelled: 10, 50, 100, 200, 400 and 800 μL by adjusting the reaction fluid level accordingly. Average dose rates were determined in the vial volume.

### In vitro clonogenic assay

For in vitro clonogenic assays the CA20948 cell line (derived from a rat pancreas tumour at our institute Erasmus MC) was used. CA208948 is a rat pancreatic tumour cell line with relative high expression of somatostatine receptor type 2 (SSTR_2_), and was cultured in DMEM supplemented with 10 % fetal calf serum (FBS, Gibco, Life technologies). Cells (500 cells/well) were seeded in 6-wellplates pre-coated with poly-l-lysine 24 h before exposure to the compounds used for labelling, except for peptide and ^213^Bi. For clonogenic assay, 400 μL of standard labelling procedure was used, and diluted in 8 mL incubation medium containing 30 mmol.L^-1^ HEPES and 0.25 % BSA. The concentration of TRIS, ascorbic acid, NaI, HCl and DTPA corresponded to 16, 3.4, 3.6, 3.6, and 6.1 × 10^−3^ mmol.L^−1^, respectively. Cells were incubated with compounds for 1 h at 37 °C in a humidified atmosphere of 5 % CO_2_ and 95 % air. Untreated CA20948 cells were used as control. After 1 h, incubation medium containing labelling compounds was removed from cells, cells were washed twice with PBS and incubated with culture medium containing 10 % FBS for 12 days. Every 2 or 3 days, culture medium was replaced by fresh medium. At day 12, colonies were fixed with 100 % ethanol and stained with hematoxyline. Survival colonies were counted and the survival CA20948 was determined. Clonogenic assay with CA20948 as function of ascorbic acid was also performed with the method described above.

## Results

### Labelling

The standard labelling protocol ^213^Bi-DOTATATE resulted in an incorporation > 99 % and a RCP > 85 % at pH 8.7 for up to 2 h after labelling. The calculated osmolarity of the labelling using the standard procedure was 0.7 Osmol.L^−1^.

**Fig. 2:**
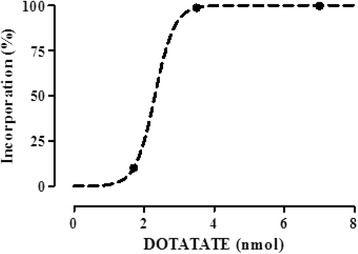
The incorporation (%) of ^213^Bi-DOTATATE 71 ± 28 MBq determined by ITLC analysis as function of peptide amount in a reaction volume of 800 μL. An S-shaped cureve has been fitted through the data with an incorporation of 50 % at 2.4 ± 0.09 nmol and a slope of 0.25 ± 0.03 nmol peptide, *n* ≥ 3

Labelling with 1.7 nmol DOTATATE in 800 μL resulted in low incorporation of 6 ± 4 % and poor RCP of 5 ± 5 % (median: 6.9 % (range: 0–8.6 %)), see Fig. 2. A high incorporation and high RCP were found when labelled with 3.5 nmol (incorporation was 99 ± 1 %, RCP was 89 ± 4 %) and 7.0 nmol peptide (incorporation was 99 ± 1 %, RCP was 88 ± 6 %).

Reducing the amount of TRIS buffer in the labelling did not show an effect on the incorporation of the labelled compound, incorporation of > 99 % and a RCP of > 85 % were found for labelling with 0.15 mol.L^−1^ and 0.34 mol.L^−1^ TRIS, corresponding to pH 8.3 and 8.7, respectively.

**Fig. 3:**
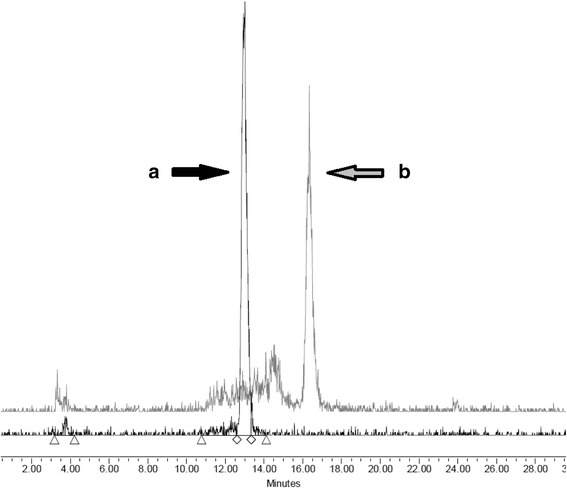
HPLC chromatogram of ^213^Bi-DOTATATE in presence **a** and absence **b** of ascorbic acid during labelling (n ≥ 3). Peak A; RT = 13.0 min and peak B; RT = 16.3 min. The y-axis represents the detected radioactive signals (mV) and x-axis represents the RT in minutes

After labelling in the presence of ascorbic acid under the standard procedure, an incorporation of >99 % and a RCP of > 85 % were found, Fig. 3a. Labelling in absence of ascorbic acid however showed a high incorporation of 99 ± 1 % but at poor RCP, < 5 % ^213^Bi-DOTATATE remained intact after labelling, as shown in the HPLC chromatogram, Fig. 3b.

**Fig. 4:**
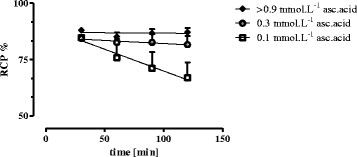
Stability as function of time of ^213^Bi-DOTATATE with different concentration ascorbic acid added during labelling, y-axis is RCP expressed in mean percentage ± SD and x-axis time in minutes after labelling (*n* ≥ 3)

To decrease the osmolarity ^213^Bi-DOTATATE labelling, labelling as function of ascorbic acid was performed, it showed that at least 0.9 mmol.L^−1^ ascorbic acid was required to maintain high incorporation and high RCP (>85 %), The labelled peptide showed a RCP > 85 % up to 2 h after labelling. A RCP of 75 ± 8 %, 83 ± 1 %, 87 ± 1 %, 86 ± 1 %, 86 ± 1 %, 87 ± 1 %, 88 ± 0.4 % were found by addition of 0.1 mmol.L^−1^, 0.3 mmol.L^−1^, 0.9 mmol.L^−1^, 2.6 mmol.L^−1^, 7.9 mmol.L^−1^, 24 mmol.L^−1^ and 71 mmol.L^−1^ ascorbic acid, respectively. Addition of less than 0.9 mmol.L^−1^ ascorbic acid resulted in high incorporation directly after radiolabeling, however the RCP decreased over time, see Fig. 4.

The adjusted labelling conditions in our experiment were 3.5–7.0 nmol peptide, in 0.15 mol.L^−1^ TRIS buffer containing 2.6 mmol.L^−1^ ascorbic acid in a reaction volume of 800 μL with 71 ± 28 MBq ^213^Bi, with a calculated osmolarity of 0.45 Osmol.L^−1^.

### Dosimetry

**Fig. 5:**
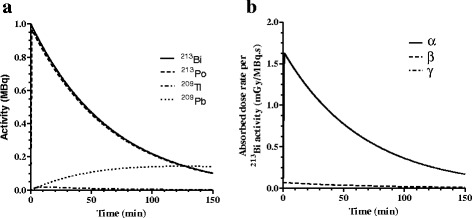
Time-integrated activity of ^213^Bi, ^213^Po, ^209^Tl and ^209^Pb determined by Bateman equations **a** and the resultant total absorbed dose rate **b** as a function of time, see Table 2. The majority of the absorbed dose rate (96 % at 60 min) is caused by the α-particles from ^213^Bi and ^213^Po

The absorbed fraction of energy from the α-particles from ^213^Bi was almost complete within the reaction fluids, 98.9 % for the 10 μL reaction volume and further rose to 99.7 % for 800 μL. The higher energy α-particles from ^213^Po showed a little more energy transfer to the vial wall; 97.2 % was absorbed in 10 μL and 99.2 % in 800 μL. The absorbed dose rate caused by ^213^Bi, ^213^Po, ^209^ Tl and ^209^Pb were predominantly contributed by the α-particles from ^213^Bi and ^213^Po, see Fig. 5a. The β^−^-particles and low energy electrons were less absorbed within the reaction fluid, e.g. the absorbed fraction of energy by β^−^-particles from ^213^Bi ranges from 44 % (10 μL) to 77 % (800 μL). The relative contribution of the β^−^-particles to the total ^213^Bi S-value thus raised from 58 to 70 %. The absorbed dose rate caused by α-particles (initially 165 mGy.s^−1^) continued to be high compared to the absorbed dose rate of β^−^-particles (initially 7 mGy.s^−1^), see Fig. 5b.

Based on the absorbed dose rate, see Table 2, the estimated absorbed dose for 100 MBq was > 230 Gy at 30 min in a reaction vial containing 800 μL hot reaction fluid. The absorbed dose was predominantly caused by the decay of α-particles, see Fig. 5b.

**Table 2:** Absorbed dose rates per unit activity (S-values, in mGy/MBq.s) in the reaction fluid for different volumes of ^213^Bi, ^213^Po, ^209^Tl and ^209^Pb. The different contributes to the S-value (α, β, low energy electrons and γ-rays) are indicated separately

^213^Bi	10 μL	50 μL	100 μL	200 μL	400 μL	800 μL
5.97 MeV α	1.94	0.39	0.19	0.097	0.049	0.024
β-particles	2.98	0.80	0.44	0.24	0.13	0.066
Auger + IC e^−^	0.20	0.048	0.026	0.013	0.0067	0.0034
γ-rays	0.0054	0.0019	0.0012	0.0007	0.0005	0.0003
*Total*	*5.12*	*1.24*	*0.67*	*0.35*	*0.18*	*0.094*
^213^Po						
8.38 MeV α	130	26.4	13.3	6.6	3.3	1.7
^209^Tl						
β-particles	3.17	0.94	0.55	0.30	0.17	0.08
Auger + IC e^−^	0.36	0.08	0.04	0.02	0.01	0.01
γ-rays	0.04	0.02	0.01	0.01	0.00	0.00
*Total*	*3.57*	*1.03*	*0.60*	*0.33*	*0.18*	*0.09*
^209^Pb						
β-particles	2.33	0.53	0.28	0.14	0.07	0.04

### Clonogenic assay

**Fig. 6:**
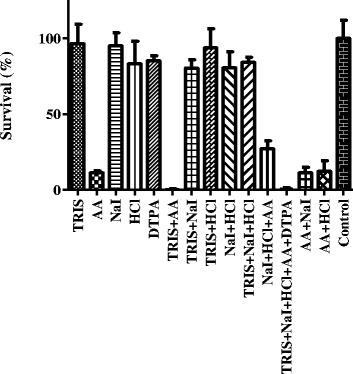
Survival (%) ± SD of CA20948 after exposure to the compounds used in standard labelling procedure, n = 3. Concentration in incubation medium was; [TRIS] = 16 mmol.L^−1^, [AA] = ascorbic acid, 3.4 mmol.L^−1^, [NaI] = 3.6 mmol.L^−1^, [HCl] = 3.6 mmol.L^−1^ and [DTPA] = 6.1 × 10^−3^ mmol.L^−1^

In all cells treated in present of ascorbic acid (corresponding to 3.4 mmol.L^−1^ in incubation medium), a significant decrease of survival was found compared to control cells, *P* < 0.05, see Fig. 6. Control cell survival was 100 % with a standard deviation of 12 %. The mean cell survival ± SD of CA20948 after exposure to ascorbic acid or in combination with TRIS, NaI, HCl and DTPA was listed in Table 3. The concentration of TRIS, NaI, HCl, and DTPA in incubation medium was 16 mmol.L^−1^, 3.6 mmol.L^−1^, 3.6 mmol.L^−1^ and 6.1 × 10^−3^ mmol.L^−1^, respectively.

**Table 3:** CA20948 clonogenic survival in mean percentage ± SD after exposure to ascorbic acid or in combination with TRIS, NaI, HCl and DTPA, *n* = 3. Concentration in incubation medium was; [TRIS] = 16 mmol.L^−1^, [AA] = ascorbic acid, 3.4 mmol.L^−1^, [NaI] = 3.6 mmol.L^−1^, [HCl] = 3.6 mmol.L^−1^ and [DTPA] = 6.1 × 10^−3^ mmol.L^−1^

Compounds	Survival %
AA	11 ± 1
TRIS + AA	1 ± 1
NaI + HCl + AA	27 ± 5
TRIS + NaI + HCl + AA + DTPA	1 ± 1
AA + NaI	11 ± 4
AA + HCl	12 ± 7

**Fig. 7:**
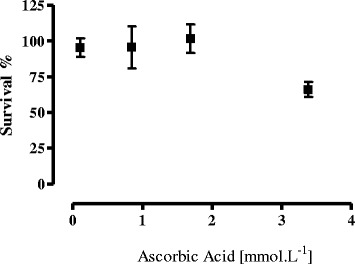
Survival (%) ± SD of CA20948 as function of concentration of ascorbic acid in DMEM incubation medium containing 30 mmol.L^-1^ HEPES and 0.25 % BSA (total volume was 8.4 mL), *n* = 3

Reducing the concentration of ascorbic acid during the labelling, resulted in an increase of cell survival, see Fig. 7.

## Discussion

A cytotoxic dose within the tumour cells is required to cause a therapeutic effect of TAT, when using a receptor-mediated process as dose delivery method. In the clinical situation using β^−^-emitters, this cytotoxic dose is in the order of 200 Gy for neuroendocrine tumours (Ilan et al. 2015; Pauwels et al. 2005) and in the preclinical situation this dose is in the order of 70 Gy for cure of CA20948 (rat pancreatic) tumour (Verwijnen et al. 2004). The limited number of receptors available on the cell membrane and relative short half-life of ^213^Bi however, require radioligands with high SA to obtain a curative tumour dose. The optimal characteristics of radioligands for TAT applications in a preclinical setting include a low injection volume, physiologic osmolarity, high SA, and suitable stability. Labelling with a radionuclide generator yielding relatively low radioactivity is a barrier for high SA radiolabelling.

The most commonly techniques used to increase the SA are purification of the elution by ion-exchange chromatography or by radiolabelling using fractionated elution to reduce the reaction volume which may improve the reaction kinetics. This may allow high incorporation yield with lower peptide amounts and thus increase the SA of the radio-peptide. To mimic this concept, ^213^Bi-DOTATATE labelling was performed with half of the elution volume to a reach lower reaction volume (total 400 μL), high incorporation yield was achieved even at reduced amount of peptide, e.g. 1.7 nmol (data not shown).

The most practical method to achieve high SA radiolabelling for preclinical applications is the use of a higher activity ^225^Ac/^213^Bi generator to accomplish the requirements needed for preclinical applications. Purification, e.g. by HPLC or solid phase extraction, might increase the SA. However, introduction of an extra purification step also increases the time interval prior to injection and thus reduces the ^213^Bi radioactivity. Moreover, HPLC purification often introduces organic compounds e.g. methanol or acetonitrile into the radiolabelling, which might be harmful to animals and limit further translation to clinical studies.

We found a correlation of ascorbic acid on cell survival; at high concentration of ascorbic acid used for labelling (71 mmol.L^−1^, corresponding to 3.4 mmol.L^−1^ in incubation medium), a significant reduction of cell survival was observed. A lower reduction of cell survival was found with only or in combinations with NaI, HCl, TRIS and DTPA compared to control cells, probably caused by the high osmolarity of NaI, HCl, TRIS or Zn-depletion to cells by DTPA (Cho et al. 2007), however this reduction was not significant. Ascorbic acid, functioning as pro-oxidant, is known to be selectively toxic, especially in vitro to certain type of tumour cells at high concentration (Park 2013). C_18_ solid phase extraction can be an option to prevent this by removing ascorbic acid from the labelling reaction. However, radiolysis continues to occur after labelling, and removing ascorbic acid might increase peptide damage caused by radiolysis. After optimizing the labelling conditions, the concentration of ascorbic acid used for labelling (2.6 mmol.L^−1^, corresponding to 0.13 mmol.L^−1^ in incubation medium) did not interfere with the outcomes of in vitro studies, therefore direct application can be performed and no purifications steps were required for further in vitro and in vivo preclinical applications.

In theory, one DOTA-peptide molecule can incorporate one atom ^213^Bi, thus at a molar ratio of 1 metal *vs* DOTA, under ideal condition the maximum theoretical SA will be 155 GBq ^213^Bi /nmol DOTA-peptide. In a study investigating the highest achievable SA, 7.0 nmol DOTATATE was labelled with different mole ratios ^209^/^213^Bi using the standard labelling procedure. At mole ratio of 0.6 ^209^/^213^Bi vs DOTATATE, the incorporation was ≥ 99 %. At mole ratio > 0.6, the incorporation started to decline, possibly caused by the presence of other cations (derived from decay product of ^213^Bi, quencher, buffer, elution solution) in the labelling reaction which interfered with the incorporation of ^213^Bi into DOTA-moiety (Breeman et al. 2005; Zhernosekov et al. 2007). In theory, a SA of 93 GBq/nmol DOTATATE (at mole ratio ^213^Bi 0.6) can be achieved under these labelling conditions.

Radiolabelling of ^213^Bi is less pH-dependent (in the range tested) as is the case of ^111^In and ^177^Lu with formation of insoluble ^111^InOCl or ^177^LuOCl (Breeman et al. 2003). Radiolabelling with ^213^Bi can be performed at pH values ranging from 4.0 to 10 (Norenberg et al. 2006; Hassfjell et al. 2003; Le Gac et al. 2011), due to the formation BiI_4_-/BiI_5_^2−^ after elution with 0.1 M/0.1 M HCl/NaI (Morgenstern et al. 2012). However, deprotonation of the carboxyl group of the DOTA appears faster at higher pH and resulting in more rapid radiolabelling kinetics (Wu et al. 1997; Szilagyi et al. 2000).

In this study, we highlighted that ITLC-SG alone as quality control is not sufficient, see labelling in absence and presence of ascorbic acid. HPLC provides beside the incorporation yield also information of peptide damage caused by α-track or radiolysis. The formation of free radicals is dependent on the type of radiation, the amount radioactivity present in the reaction mixture and on the duration of exposure, all of which have been shown to induce radiation damage of the peptide. Quenchers such as gentisic acid in combination with ascorbic acid and ethanol are often used to prevent radiolysis in radiolabelling with ^111^In or ^177^Lu (de Blois et al. 2012). Radiolysis leads to damage of peptide and will decrease the RCP.

With α-radiation more water radicals, such as peroxide, will be formed besides the direct radiation damage from the high LET α-particle tracks (Deutsch 1998). Stanton et al. observed more biological efficacy on SV40 viral DNA in vitro caused by low LET compared to high LET and Sugo et al. demonstrated less degradation of TODGA, a tridentate complexing agent, after irradiation with helium ion beam than that by that of γ-ray irradiation (Sugo 2009; Stanton et al. 1993). Since the activated radicals are formed along the α-particle tracks and therefore decreasing the probability for damage by both the direct and the radical induced effect in the reaction solution, thus a lower amount of quenchers is required to prevent radical damage to the peptide. In our case, ascorbic acid alone showed sufficient protection capacity to prevent damage to the peptide, so no other quenchers were introduced to the labelling, also to prevent further increase of the osmolarity. Higher osmolarity would require further diluting of the injection matrix for small-animals applications, leading to lower radioactivity injections for effective TAT in small animals.

Here we present detailed results obtained with MCNP calculations of the absorbed dose of ^213^Bi in the reaction vial. The calculated absorbed dose is an average absorbed dose estimated in a vial of 800 μL, mostly caused by α-particles, but also including doses from β^−^-particles and γ-rays emitted by the decay daughters of ^213^Bi. As shown in Fig. 4, 0.9 mmol.L^−1^ ascorbic acid was required to protect the peptide in a vial containing 71 ± 28 MBq in 800 μL, with an absorbed dose of ~200 Gy. At higher radioactivities of ^213^Bi or smaller reaction volumes, more ascorbic acid was required to protect the peptide, since the absorbed dose in the reaction vial increased and formation of radicals also increased. These doses are much lower than the absorbed doses considered to cause radiation damage in DTPA and TODGA, which are in the order of 100 kGy (Sugo 2009; Stanton et al. 1993). The main sensitive region for radiation damage will therefore be in the DOTATATE’s disulfide bond and its other ionized parts, as the DOTA cage may be considered not to be damaged at the “low” absorbed doses considered.

The activation energy for the DOTA ring inversion process is in the order of 65 kJ/mol (Lima et al. 2015). The initial dose rate by the α-radiation in the vial with 100 MBq ^213^Bi amounts 165 mGy/s, which corresponds to an absorbed energy rate of 0.13 mJ/s in 800 μL with 7 nmol compound, or 19 kJ/mol.s. This indicative calculation shows that the momentary dose rate is on average not energetic enough to create configuration changes within the DOTA ring. The energy of 65 kJ/mol is reached within 3.5 s.

Stability, incorporation yield and SA of ^213^Bi-labelled peptides are influenced by the affinity/stability of the metal-chelator complex, radiolysis or by the recoil effect both during and after labelling. High LET α-particles cause reactive hydrogen radicals in an aqueous environment (Bibler 1972). These radicals damage the peptide, which can lead to losses of affinity of the peptide to its receptor. The relative high recoil energy of ^213^Po, 160 keV, is a critical challenge for the choice of chelator, since complexation of metal and chelator is dependent on the binding energy. This complex should remain stable until reaching the targeted receptor. DOTA (with a Log K of 30.3) is not considered to be the most suitable chelator for ^213^Bi, due to relative poor labelling kinetics compared to derivatives of DTPA, e.g. CHX-A-DTPA (Brechbiel 2001; Stavila et al. 2006). Nevertheless, we demonstrated that ^213^Bi formed a highly stable complex with DOTA in a relative short radiolabelling time of 5 min. Furthermore, under optimized radiolabelling conditions, the labelling remained stable up to 2 h after labelling.

## Conclusion

The optimized ^213^Bi labelling conditions demonstrated to be suitable for labelling of DOTATATE and proofed ready-to-use for preclinical applications. The labelling procedure presented herein resulted in high incorporation yield, high RCP and high stability up to 2 h after labelling. The addition of quenchers such as ascorbic acid during labelling appeared essential for the protection of the peptide, since the absorbed dose rate within the reaction was high; > 165 mGy.s^−1^ with 100 MBq of ^213^Bi.

## Additional files

Additional file 1: Bateman equations used to determine the activity of ^213^Bi, ^213^Po, ^209^ Tl and ^209^Pb. (DOCX 17 kb)

## References

[CR1] Bergsma H, van Vliet EI, Teunissen JJ, Kam BL, de Herder WW, Peeters RP (2012). Peptide receptor radionuclide therapy (PRRT) for GEP-NETs. Best Pract Res Clin Gastroenterol.

[CR2] Bibler NE (1972). Gamma and alpha radiolysis of aqueous solutions of diethylenetriaminepenta acetic acid. J Inorg Nucl Chem.

[CR3] Bodei L, Cremonesi M, Grana CM, Chinol M, Baio SM, Severi S (2012). Yttrium-labelled peptides for therapy of NET. Eur J Nucl Med Mol Imaging.

[CR4] Brechbiel MW (2001). Chelated metal ions for therapeutic and diagnostic applications. Exp Biol Med (Maywood).

[CR5] Breeman WA, Kwekkeboom DJ, Kooij PP, Bakker WH, Hofland LJ, Visser TJ (1995). Effect of dose and specific activity on tissue distribution of indium-111-pentetreotide in rats. J Nucl Med.

[CR6] Breeman WA, de Jong M, Kwekkeboom DJ, Valkema R, Bakker WH, Kooij PP (2001). Somatostatin receptor-mediated imaging and therapy: basic science, current knowledge, limitations and future perspectives. Eur J Nucl Med.

[CR7] Breeman WA, De Jong M, Visser TJ, Erion JL, Krenning EP (2003). Optimising conditions for radiolabelling of DOTA-peptides with 90Y, 111In and 177Lu at high specific activities. Eur J Nucl Med Mol Imaging.

[CR8] Breeman WA, de Jong M, de Blois E, Bernard BF, Konijnenberg M, Krenning EP (2005). Radiolabelling DOTA-peptides with 68Ga. Eur J Nucl Med Mol Imaging.

[CR9] Cho YE, Lomeda RA, Ryu SH, Lee JH, Beattie JH, Kwun IS (2007). Cellular Zn depletion by metal ion chelators (TPEN, DTPA and chelex resin) and its application to osteoblastic MC3T3-E1 cells. Nutr Res Pract.

[CR10] Cordier D, Forrer F, Bruchertseifer F, Morgenstern A, Apostolidis C, Good S (2010). Targeted alpha-radionuclide therapy of functionally critically located gliomas with 213Bi-DOTA-[Thi8, Met (O2) 11]-substance P: a pilot trial. Eur J Nucl Med Mol Imaging.

[CR11] de Blois E, Sze Chan H, Naidoo C, Prince D, Krenning EP, Breeman WA (2011). Characteristics of SnO2-based 68Ge/68Ga generator and aspects of radiolabelling DOTA-peptides. Appl Radiat Isot.

[CR12] de Blois E, Chan HS, Konijnenberg M, de Zanger R, Breeman WA (2012). Effectiveness of quenchers to reduce radiolysis of (111) in- or (177) Lu-labelled methionine-containing regulatory peptides. Maintaining radiochemical purity as measured by HPLC. Curr Top Med Chem.

[CR13] de Jong M, Breeman WA, Bernard BF, van Gameren A, de Bruin E, Bakker WH (1999). Tumour uptake of the radiolabelled somatostatin analogue [DOTA0, TYR3] octreotide is dependent on the peptide amount. Eur J Nucl Med.

[CR14] Deutsch JC (1998). Ascorbic acid oxidation by hydrogen peroxide. Anal Biochem.

[CR15] Eckerman KF. MIRD2008.

[CR16] Hassfjell S, Kongshaug KO, Romming C. Synthesis, crystal structure and chemical stability of bismuth (III) complexed with 1,4,7,10-tetraazacyclododecane-1,4,7,10-tetramethylene phosphonic acid (H8DOTMP). Dalton T. 2003;(7):1433–7.

[CR17] Hendricks JS. MCNPX Extensions Version 2.5.02005.

[CR18] Ilan E, Sandstrom M, Wassberg C, Sundin A, Garske-Roman U, Eriksson B (2015). Dose response of pancreatic neuroendocrine tumors treated with peptide receptor radionuclide therapy using 177Lu-DOTATATE. J Nucl Med.

[CR19] Jurcic JG, Caron PC, Nikula TK, Papadopoulos EB, Finn RD, Gansow OA (1995). Radiolabeled anti-CD33 monoclonal antibody M195 for myeloid leukemias. Cancer res.

[CR20] Konijnenberg MW (2014). Therapeutic application of CCK2R-targeting PP-F11: influence of particle range, activity and peptide amount. Eur J Nucl Med Mol Imaging Res.

[CR21] Kratochwil C, Giesel FL, Bruchertseifer F, Apostolidis C, Mier W, Morgenstern A (2011). Dose escalation study of peptide receptor alpha-therapy with arterially administered Bi-213-DOTATOC in GEP-NET patients refractory to beta-emitters. Eur J Nucl Med Mol Imaging.

[CR22] Kratochwil C, Giesel FL, Bruchertseifer F, Mier W, Apostolidis C, Boll R (2014). (2) (1) (3) Bi-DOTATOC receptor-targeted alpha-radionuclide therapy induces remission in neuroendocrine tumours refractory to beta radiation: a first-in-human experience. Eur J Nucl Med Mol Imaging.

[CR23] Le Gac S, Najjari B, Motreff N, Remaud-Le Saec P, Faivre-Chauvet A, Dimanche-Boitrel MT (2011). Unprecedented incorporation of alpha-emitter radioisotope 213Bi into porphyrin chelates with reference to a daughter isotope mediated assistance mechanism. Chem Commun (Camb).

[CR24] Lima LM, Beyler M, Delgado R, Platas-Iglesias C, Tripier R (2015). Investigating the complexation of the Pb (2+)/Bi (3+) pair with dipicolinate cyclen ligands. Inorg Chem.

[CR25] Ma D, McDevitt MR, Finn RD, Scheinberg DA (2001). Breakthrough of 225Ac and its radionuclide daughters from an 225Ac/213Bi generator: development of new methods, quantitative characterization, and implications for clinical use. Appl Radiat Isot.

[CR26] McDevitt MR, Finn RD, Sgouros G, Ma D, Scheinberg DA (1999). An 225Ac/213Bi generator system for therapeutic clinical applications: construction and operation. Appl Radiat Isot.

[CR27] MCNP-Team. The Monte Carlo codes MCNP52005.

[CR28] Morgenstern A, Bruchertseifer F, Apostolidis C (2012). Bismuth-213 and actinium-225—generator performance and evolving therapeutic applications of two generator-derived alpha-emitting radioisotopes. Curr Radiopharm.

[CR29] Norenberg JP, Krenning BJ, Konings IR, Kusewitt DF, Nayak TK, Anderson TL (2006). 213Bi-[DOTA0, Tyr3] octreotide peptide receptor radionuclide therapy of pancreatic tumors in a preclinical animal model. Clin Cancer Res.

[CR30] Park S (2013). The effects of high concentrations of vitamin C on cancer cells. Nutrients.

[CR31] Pauwels S, Barone R, Walrand S, Borson-Chazot F, Valkema R, Kvols LK (2005). Practical dosimetry of peptide receptor radionuclide therapy with (90) Y-labeled somatostatin analogs. J Nucl Med.

[CR32] Song H, Shahverdi K, Huso DL, Esaias C, Fox J, Liedy A (2008). 213Bi (alpha-emitter)-antibody targeting of breast cancer metastases in the neu-N transgenic mouse model. Cancer Res.

[CR33] Stanton J, Taucher-Scholz G, Schneider M, Heilmann J, Kraft G (1993). Protection of DNA from high LET radiation by two OH radical scavengers, tris (hydroxymethyl) aminomethane and 2-mercaptoethanol. Radiat Environ Biophys.

[CR34] Stavila V, Davidovich RL, Gulea A, Whitmire KH (2006). Bismuth (III) complexes with aminopolycarboxylate and polyaminopolycarboxylate ligands: chemistry and structure. Coordin Chem Rev.

[CR35] Sugo Y. Radiolysis study of actinide complexing agent by irradiation with helium ion beam. Radiat Phys Chem. 2009;78.

[CR36] Szilagyi E, Toth E, Kovacs Z, Platzek J, Raduchel B, Brucher E (2000). Equilibria and formation kinetics of some cyclen derivative complexes of lanthanides. Inorg Chim Acta.

[CR37] van der Zwan WA, Bodei L, Mueller-Brand J, de Herder WW, Kvols LK, Kwekkeboom DJ (2015). GEPNETs UPDATE, radionuclide therapy in neuroendocrine tumors. Eur J Endocrinol.

[CR38] Verwijnen S, Capello A, Bernard B, van den Aardweg G, Konijnenberg M, Breeman W (2004). Low-dose-rate irradiation by 131I versus high-dose-rate external-beam irradiation in the rat pancreatic tumor cell line CA20948. Cancer Biother Radiopharm.

[CR39] Wild D, Frischknecht M, Zhang H, Morgenstern A, Bruchertseifer F, Boisclair J (2011). Alpha- versus beta-particle radiopeptide therapy in a human prostate cancer model (213Bi-DOTA-PESIN and 213Bi-AMBA versus 177Lu-DOTA-PESIN). Cancer Res.

[CR40] Wu SL, Johnson KA, Horrocks WD (1997). Kinetics of formation of Ca2+ complexes of acyclic and macrocyclic poly (amino carboxylate) ligands: bimolecular rate constants for the fully-deprotonated ligands reveal the effect of macrocyclic ligand constraints on the rate-determining conversions of rapidly-formed intermediates to the final complexes. Inorg Chem.

[CR41] Zhernosekov KP, Filosofov DV, Baum RP, Aschoff P, Bihl H, Razbash AA (2007). Processing of generator-produced 68Ga for medical application. J Nucl Med.

