# Supplementary material for: Optimizing labelling conditions of 213Bi-DOTATATE for preclinical applications of peptide receptor targeted alpha therapy
Source: EJNMMI Radiopharm Chem. 2016 May 14;1:9. doi: 10.1186/s41181-016-0014-4 (PMC5843812; doi:10.1186/s41181-016-0014-4)
Supplement: Supplementary file 1 — Bateman equations used to determine the activity of 213Bi, 213Po, 209 Tl and 209Pb. (DOCX 17 kb) [file 41181_2016_14_MOESM1_ESM.docx]

**Supporting information**

**Optimizing labelling conditions of ^213^Bi-DOTATATE for preclinical applications of peptide receptor targeted alpha therapy**

Ho Sze Chan^1^, Erik de Blois^1^, Mark W. Konijnenberg^1^, Alfred Morgenstern^2^, Frank Bruchertseifer^2^, Jeffrey P. Norenberg^3^, Fred J. Verzijlbergen^1^, Marion de Jong^1^, Wouter A. P. Breeman^1^

^1^ Erasmus MC, Department of Radiology and Nuclear Medicine, Rotterdam, the Netherlands

^2^ European Commission, Joint Research Centre, Institute for Transuranium Elements (ITU), Karlsruhe, Germany

^3^ Radiopharmaceutical Sciences Program, College of Pharmacy, University of New Mexico Health Sciences Center, Albuquerque, NM, United States

**Bateman equations used to determine the activity of ^213^Bi, ^213^Po, ^209^ Tl and ^209^Pb.**

*Equation 1.*

$$A_{Bi}\left( t \right)=A_{Bi}\left( 0 \right)exp\left( \lambda_{Bi}t \right)$$

$$A_{Po}\left( t \right)=\frac{{BR}_{Po}\lambda_{Po}A_{Bi}(0)}{\lambda_{Bi}-\lambda_{Po}}\left( \exp\left( {-\lambda}_{Po}t \right)-exp(-\lambda_{Bi}t) \right)\approx{BR}_{Po}A_{Bi}(0)exp(-\lambda_{Bi}t)$$

$$A_{Tl}\left( t \right)=\frac{{BR}_{Tl}\lambda_{Tl}}{\lambda_{Bi}-\lambda_{Tl}}A_{Bi}(0)\left( \exp\left( {-\lambda}_{Tl}t \right)-exp(-\lambda_{Bi}t) \right)$$

$A_{\mathrm{Pb}}\left( t \right)=\frac{\mathrm{BR}_{\mathrm{Po}}\lambda_{\mathrm{Pb}}}{\lambda_{\mathrm{Bi}}-\lambda_{\mathrm{Pb}}}A_{\mathrm{Bi}}(0)\left( \exp\left( -\lambda_{\mathrm{Pb}}t \right)-\exp\left( -\lambda_{\mathrm{Bi}}t \right) \right)-\frac{\mathrm{BR}_{\mathrm{Tl}}\lambda_{\mathrm{Tl}}\lambda_{\mathrm{Pb}}}{\lambda_{\mathrm{Bi}}-\lambda_{\mathrm{Tl}}}A_{\mathrm{Bi}}(0)\left( \frac{\exp\left( -\lambda_{\mathrm{Pb}}t \right)-exp \left( {-\lambda}_{\mathrm{Tl}}t \right)}{\lambda_{\mathrm{Tl}}-\lambda_{\mathrm{Pb}}}-\frac{\exp\left( -\lambda_{\mathrm{Pb}}t \right)-\exp\left( -\lambda_{\mathrm{Bi}}t \right)}{\lambda_{\mathrm{Bi}}-\lambda_{\mathrm{Pb}}} \right)$

$$A_{\mathrm{Pb}}\left( t \right)=$$

$$\frac{\mathrm{BR}_{\mathrm{Po}}\lambda_{\mathrm{Pb}}}{\lambda_{\mathrm{Bi}}-\lambda_{\mathrm{Pb}}}A_{\mathrm{Bi}}\left( 0 \right)\left( \frac{\exp\left( -\lambda_{\mathrm{Pb}}t \right)-exp \left( {-\lambda}_{\mathrm{Po}}t \right)}{\lambda_{\mathrm{Po}}-\lambda_{\mathrm{Pb}}}-\frac{\exp\left( -\lambda_{\mathrm{Pb}}t \right)-\exp\left( -\lambda_{\mathrm{Bi}}t \right)}{\lambda_{\mathrm{Bi}}-\lambda_{\mathrm{Pb}}} \right)$$

$-\frac{\mathrm{BR}_{\mathrm{Tl}}\lambda_{\mathrm{Tl}}\lambda_{\mathrm{Pb}}}{\lambda_{\mathrm{Bi}}-\lambda_{\mathrm{Tl}}}A_{\mathrm{Bi}}(0)\left( \frac{\exp\left( -\lambda_{\mathrm{Pb}}t \right)-exp \left( {-\lambda}_{\mathrm{Tl}}t \right)}{\lambda_{\mathrm{Tl}}-\lambda_{\mathrm{Pb}}}-\frac{\exp\left( -\lambda_{\mathrm{Pb}}t \right)-\exp\left( -\lambda_{\mathrm{Bi}}t \right)}{\lambda_{\mathrm{Bi}}-\lambda_{\mathrm{Pb}}} \right)$ $A_{\mathrm{Pb}}\left( t \right)=\frac{\mathrm{BR}_{\mathrm{Po}}\lambda_{\mathrm{Pb}}}{\lambda_{\mathrm{Bi}}-\lambda_{\mathrm{Pb}}}A_{\mathrm{Bi}}(0)\left( \exp\left( -\lambda_{\mathrm{Pb}}t \right)-\exp\left( -\lambda_{\mathrm{Bi}}t \right) \right)-\frac{\mathrm{BR}_{\mathrm{Tl}}\lambda_{\mathrm{Tl}}\lambda_{\mathrm{Pb}}}{\lambda_{\mathrm{Bi}}-\lambda_{\mathrm{Tl}}}A_{\mathrm{Bi}}(0)\left( \frac{\exp\left( -\lambda_{\mathrm{Pb}}t \right)-exp \left( {-\lambda}_{\mathrm{Tl}}t \right)}{\lambda_{\mathrm{Tl}}-\lambda_{\mathrm{Pb}}}-\frac{\exp\left( -\lambda_{\mathrm{Pb}}t \right)-\exp\left( -\lambda_{\mathrm{Bi}}t \right)}{\lambda_{\mathrm{Bi}}-\lambda_{\mathrm{Pb}}} \right)$

$A_{\mathrm{Pb}}\left( t \right)=\frac{\mathrm{BR}_{\mathrm{Po}}\lambda_{\mathrm{Pb}}}{\lambda_{\mathrm{Bi}}-\lambda_{\mathrm{Pb}}}A_{\mathrm{Bi}}(0)\left( \exp\left( -\lambda_{\mathrm{Pb}}t \right)-\exp\left( -\lambda_{\mathrm{Bi}}t \right) \right)-\frac{\mathrm{BR}_{\mathrm{Tl}}\lambda_{\mathrm{Tl}}\lambda_{\mathrm{Pb}}}{\lambda_{\mathrm{Bi}}-\lambda_{\mathrm{Tl}}}A_{\mathrm{Bi}}(0)\left( \frac{\exp\left( -\lambda_{\mathrm{Pb}}t \right)-exp \left( {-\lambda}_{\mathrm{Tl}}t \right)}{\lambda_{\mathrm{Tl}}-\lambda_{\mathrm{Pb}}}-\frac{\exp\left( -\lambda_{\mathrm{Pb}}t \right)-\exp\left( -\lambda_{\mathrm{Bi}}t \right)}{\lambda_{\mathrm{Bi}}-\lambda_{\mathrm{Pb}}} \right)$
